# Adolescent idiopathic scoliosis non-surgical interventions: a systematic review and network meta-analysis of Cobb angle and SRS-22 outcomes

**DOI:** 10.3389/fped.2026.1908709

**Published:** 2026-09-09

**Authors:** Shaobo Yang, Feihong Cai, Ruixuan Xie, Jing Li, Jun Zhao, Chen Zhang, Dong Hou, Kai Li, Han Leng, Xiaohui Dou, Jing Zhang, Xiaojuan Cui, Juan Wang, Xiaoyun Yuan, Haitao Zhang, Dezhi Tang, Jiantao Wen, Jin Huang

**Affiliations:** 1 School of Integrated Chinese and Western Medicine, Gansu University of Chinese Medicine, Lanzhou, China; 2 Institute of Spine Diseases, Longhua Hospital, Shanghai University of Traditional Chinese Medicine, Shanghai, China; 3 School of Ophthalmology and Optometry (School of Biomedical Engineering), Wenzhou Medical University, Wenzhou, China; 4 Department of Spleen and Stomach Diseases, Gansu Provincial Hospital of Traditional Chinese Medicine, Lanzhou, China; 5 Department of Orthopedics, Gansu Provincial Hospital of Traditional Chinese Medicine, Lanzhou, China; 6 Department of Spine Orthopedics, Gansu Provincial Hospital of Traditional Chinese Medicine, Lanzhou, China; 7 Department of Emergency Surgery, The First People's Hospital of Longnan, Longnan, China; 8 School of Clinical Chinese Medicine, Gansu University of Chinese Medicine, Lanzhou, China; 9 Department of Artificial Joint and Knee Surgery, The First Affiliated Hospital of Guangzhou University of Chinese Medicine, Guangzhou, China

**Keywords:** adolescent idiopathic scoliosis, bracing, Cobb angle, exercise therapy, network meta-analysis, non-surgical treatment, SRS-22

## Abstract

**Background:**

Adolescent idiopathic scoliosis (AIS) is a common three-dimensional spinal deformity. Non-surgical interventions are important for controlling curve progression and maintaining quality of life, but their comparative effectiveness remains uncertain.

**Objective:**

To compare the effects of commonly used non-surgical interventions for AIS on Cobb angle and SRS-22 outcomes through a systematic review and network meta-analysis.

**Methods:**

PubMed/MEDLINE, Embase, Web of Science, Cochrane Library, and major Chinese databases were searched from inception to April 4, 2026. Randomized controlled trials (RCTs) comparing non-surgical interventions and reporting Cobb angle or SRS-22 were included for the primary network meta-analysis. Prospective non-randomized studies served as supplementary evidence. Effect sizes were expressed as standardized mean differences (SMDs) with 95% confidence intervals (CIs). Ranking was assessed using SUCRA, interpreted cautiously with heterogeneity and evidence sparsity.

**Results:**

Thirteen comparative studies (709 AIS subjects) were included (10 RCTs, 3 non-randomized). Interventions included Schroth training, bracing, scoliosis-specific exercises, core stabilization, and traditional Chinese medicine rehabilitation. Traditional pairwise meta-analysis showed a pooled SMD of −1.39 (95% CI: −2.20 to −0.59) with high heterogeneity (I^2^ = 95.1%). Network ranking suggested that bracing and Schroth-related therapies had a trend toward better short-term Cobb angle control, but most estimates were imprecise and the evidence network was sparse. Evidence on SRS-22 was limited and exploratory.

**Conclusion:**

Some non-surgical interventions may improve Cobb angle in AIS; bracing and Schroth-related therapies show a trend toward relative superiority for short-term Cobb angle control. However, due to sparse networks, high heterogeneity, and potential small-study effects, results should be interpreted cautiously and not solely based on SUCRA rankings. SRS-22 evidence remains insufficient. High-quality RCTs with longer follow-up are needed.

**Systematic Review Registration:**

PROSPERO CRD42024546325.

## 1 Introduction

Adolescent idiopathic scoliosis (AIS) is a three-dimensional spinal deformity occurring during adolescence, typically defined by a coronal Cobb angle of ≥10°, often accompanied by vertebral rotation, trunk asymmetry, and postural abnormalities (1). AIS is one of the most common types of spinal deformities in adolescents, with a prevalence of approximately 1%–3%, and a relatively higher incidence in females. For patients still in the skeletal growth phase, the curve has a potential risk of further progression, which can lead to trunk deformity, impaired self-image, and psychological distress. When the curve progresses to a more severe degree, it may also cause pain, functional limitations, and even impact cardiopulmonary function. Therefore, timely identification and effective non-surgical interventions to control curve progression, improve spinal alignment, and maintain health-related quality of life before skeletal maturity are important goals in the clinical management of AIS.

According to current conservative treatment concepts and relevant guidelines, non-surgical treatment is generally indicated for patients with mild-to-moderate AIS, particularly those with a Cobb angle of approximately 10°–45° who still have growth potential. When the curve progresses to a severe degree or reaches the surgical threshold, patients may require spinal corrective surgery (2). Although surgical treatment is valuable for correcting severe deformities, it carries risks of trauma, complications, higher medical costs, and issues related to long-term internal fixation (3). Therefore, optimizing effective interventions during the conservative treatment phase to potentially delay or avoid curve progression to the surgical threshold remains a key clinical issue in AIS prevention and treatment research.

Currently, non-surgical interventions for AIS are diverse, mainly including bracing, Schroth training, scoliosis-specific exercises (SSE), core stabilization training, general exercise therapy, and traditional Chinese medicine (TCM)-related rehabilitation interventions (4, 5). Bracing is primarily used for patients who are still in the growth period and at risk of progression, with its effectiveness closely related to wearing time, compliance, baseline curve severity, and skeletal maturity. Schroth training and other scoliosis-specific exercises emphasize three-dimensional self-correction, postural control, rotational breathing, and neuromuscular re-education, aiming to improve trunk asymmetry and enhance patients’ active control abilities. Core stabilization training, traditional exercise, and TCM-related rehabilitation methods are also used in AIS conservative management in some regions or clinical scenarios. However, significant variations exist across studies in intervention content, treatment intensity, intervention duration, follow-up time, patient baseline characteristics, and outcome assessments, leading to a lack of consensus on the comparative efficacy of different non-surgical interventions.

Previous research has often focused on direct comparisons between a single intervention and a control treatment. While traditional pairwise meta-analysis can synthesize evidence for specific comparisons, it is difficult to simultaneously compare multiple treatments within the same framework or leverage indirect evidence when direct head-to-head comparisons are lacking. Network meta-analysis (NMA) can integrate direct and indirect evidence, compare multiple interventions within a single statistical model, and provide supplementary information for clinical decision-making through ranking probabilities. However, the interpretation of NMA must be based on reasonable node definitions, acceptable clinical homogeneity, transitivity assumptions, and consistency between direct and indirect evidence. Especially in the field of AIS non-surgical treatment, where intervention protocols are complex, implementation methods vary significantly, and evidence for some outcomes is sparse, treatment rankings should not be simplistically interpreted as deterministic efficacy hierarchies but rather judged comprehensively in conjunction with effect estimates, confidence intervals, heterogeneity, risk of bias, and evidence quality (6, 7).

Furthermore, research on non-surgical AIS treatment should not only focus on radiographic outcomes but also emphasize patient-reported outcomes. The Cobb angle is a core radiographic indicator for assessing curve severity and treatment response, but its change does not necessarily equate to improvements in quality of life, physical function, or self-image. The SRS-22, a commonly used health-related quality of life assessment tool for scoliosis patients, reflects subjective patient benefits in terms of pain, function, mental health, self-image, and treatment satisfaction. Therefore, evaluating both Cobb angle and SRS-22 outcomes concurrently facilitates a more comprehensive understanding of the clinical value of non-surgical interventions for AIS patients.

Based on this background, this study employed a systematic review and network meta-analysis to systematically compare the effects of commonly used non-surgical interventions for AIS on Cobb angle and SRS-22 outcomes. This study used randomized controlled trials as the evidence base for the primary network meta-analysis and included prospective non-randomized comparative studies as supplementary evidence for result interpretation and robustness assessment. The study aimed to synthesize existing comparative evidence, evaluate the relative effects of different non-surgical interventions on radiographic and patient-reported outcomes, and provide evidence-based references for optimizing conservative treatment strategies for AIS, while considering evidence sparsity, heterogeneity, and methodological limitations.

## 2 Methods

### 2.1 Study design and reporting standards

This study is a systematic review and network meta-analysis. The study protocol was registered with the International Prospective Register of Systematic Reviews (PROSPERO) under registration number CRD42024546325. This study was designed, conducted, and reported according to the PRISMA extension statement for network meta-analyses (PRISMA-NMA) (8). This study aimed to compare the relative effects of commonly used non-surgical interventions for adolescent idiopathic scoliosis (AIS) on Cobb angle and Scoliosis Research Society-22 (SRS-22) questionnaire outcomes.

Given the limited number of direct head-to-head comparison studies in the field of AIS non-surgical treatment, this systematic review included both randomized controlled trials and prospective non-randomized comparative studies. To ensure the internal validity of the primary effect estimates, the primary network meta-analysis included only randomized controlled trials; prospective non-randomized comparative studies were not included in the primary network estimation but served as supplementary evidence for result interpretation and robustness assessment.

### 2.2 Search strategy

Systematic searches were conducted in PubMed/MEDLINE, Embase, Web of Science, Cochrane Library, and major Chinese databases, including China National Knowledge Infrastructure (CNKI), Wanfang Database, and VIP Database. The search period covered from the inception of each database to April 4, 2026. The search strategy combined MeSH terms and free-text words, adapted according to the indexing rules of each database.

English search terms included the following terms and their combinations: “Adolescent Idiopathic Scoliosis,” “AIS,” “Scoliosis,” “Exercise Therapy,” “Physiotherapeutic Scoliosis-Specific Exercises,” “PSSE,” “Schroth,” “Scientific Exercise Approach to Scoliosis,” “SEAS,” “Core Stabilization,” “Physical Activity,” “Rehabilitation,” “Traditional Chinese Medicine,” “Acupuncture,” “Tuina,” “Massage,” “Guasha,” “Cupping,” “Daoyin,” “Qigong,” “Tai Chi,” “Orthosis,” “Brace,” and “Bracing.” Chinese search terms included “青少年特发性脊柱侧凸,” “青少年特发性脊柱侧弯,” “运动疗法,” “脊柱侧凸特异性运动,” “Schroth,” “支具,” “中医,” “针刺,” “推拿,” “导引,” “气功,” “太极,” etc. During the search process, reference lists of included studies and relevant reviews were manually screened to identify potentially missed studies. The full search strategy can be provided as supplementary material according to journal requirements.

### 2.3 Eligibility criteria

#### 2.3.1 Study types

Clinical comparative studies comparing at least two non-surgical interventions for AIS, or comparing a non-surgical intervention with a usual care control, were included. The primary network meta-analysis included only randomized controlled trials, including parallel-group RCTs, assessor-blinded randomized trials, single-blind RCTs, and feasibility RCTs. Prospective non-randomized comparative studies, including prospective controlled cohort studies, comprehensive cohort studies, and prospective observational controlled studies, were included only as supplementary evidence in the systematic review and were not used for effect estimation in the primary network meta-analysis.

#### 2.3.2 Participants

Participants diagnosed with AIS, typically aged 10–18 years with a Cobb angle range of 10°–45°, were included. If the study participants were predominantly within the adolescent age range and the original study explicitly described the condition as AIS, studies including a small number of participants under 10 years of age were retained. Included studies were not restricted by participant sex, race, nationality, disease duration, or curve type; however, these characteristics were extracted to assess clinical heterogeneity and the plausibility of the transitivity assumption.

#### 2.3.3 Interventions

Included non-surgical interventions comprised, but were not limited to, the following types: (1) exercise therapy, including Schroth training, SSE, SEAS, Dobomed, three-dimensional exercise therapy, core stabilization training, Pilates, yoga, and general physical activity; (2) bracing, including Boston brace, Milwaukee brace, Charleston brace, Providence brace, and other orthotic braces; (3) traditional Chinese medicine (TCM)-related rehabilitation interventions, including tuina (massage), acupuncture, electroacupuncture, warm needling, moxibustion, cupping, guasha, daoyin, tai chi, qigong, and related comprehensive treatments; (4) combined interventions, including exercise combined with bracing, TCM-related rehabilitation combined with exercise, or other multimodal non-surgical treatments. Control measures included observation, usual care, health education, general physical activity, no treatment, or other non-surgical control protocols.

#### 2.3.4 Outcomes

Included studies were required to report at least one of the following outcomes: (1) Cobb angle, as the primary indicator for assessing radiographic severity of the curve and treatment response, reported either as post-treatment final values or change from baseline to post-treatment; (2) SRS-22 total score, as a patient-reported outcome for health-related quality of life. If a study reported both SRS-22 domain scores and total score, the total score was preferentially extracted; due to inconsistent reporting of domain scores across studies, separate network meta-analysis for SRS-22 domain scores was not performed. If other quality of life scales were reported, they were used only for descriptive summarization and not included in the primary quantitative synthesis.

#### 2.3.5 Exclusion criteria

The following studies were excluded: (1) duplicate publications; (2) reviews, meta-analyses, case reports, case series, conference abstracts, commentaries, letters, animal studies, *in vitro* experiments, and non-clinical studies; (3) studies where participants were not AIS patients or where the Cobb angle did not meet inclusion criteria and data for eligible participants could not be extracted separately; (4) studies involving surgical treatment, or where non-surgical interventions could not be clearly categorized; (5) studies not reporting Cobb angle or SRS-22 outcomes, or where no usable data for analysis could be obtained; (6) non-comparative studies without a concurrent control group.

### 2.4 Study selection and data extraction

Search results were imported into EndNote reference management software, and duplicate records were removed. Two investigators independently screened titles and abstracts to preliminarily exclude obviously ineligible records. Subsequently, full texts of potentially eligible studies were obtained and assessed against the pre-specified eligibility criteria. Disagreements between the two investigators were first resolved through discussion; if consensus could not be reached, a third investigator made the final decision.

Data were extracted using a pre-designed data extraction form. Two investigators independently extracted the following information: first author, publication year, country or region, study design, sample size, participant age, sex, baseline Cobb angle, skeletal maturity indicators (e.g., Risser sign, menarcheal status), curve type, intervention, control measure, treatment frequency, treatment intensity, intervention duration, follow-up duration, co-interventions, Cobb angle data, SRS-22 total score, and information required for risk of bias assessment. After data extraction, the two investigators cross-checked the extracted data; discrepancies were resolved through discussion or by referring back to the original publication.

For studies with multiple follow-up time points, outcome data at the end of treatment or closest to the end of treatment were preferentially extracted; if studies reported longer-term follow-up results, the follow-up duration was recorded, and the impact of follow-up differences on effect estimates was considered during result interpretation. If studies reported both change scores and post-treatment final values, change scores were preferentially extracted; if only post-treatment final values were reported, final values were extracted for analysis. For studies reporting only medians, ranges, or interquartile ranges, established statistical methods were used to estimate means and standard deviations where appropriate. If standard deviations were not directly reported, they were calculated from standard errors, confidence intervals, *p*-values, or other available statistics; if still unattainable, cautious imputation based on clinically and methodologically similar studies was performed, and the potential impact was considered during result interpretation.

### 2.5 Intervention node definitions

To clarify the intended purposes of the exercise nodes: Schroth-based and other scoliosis-specific exercises aim to improve three-dimensional spinal alignment, trunk symmetry, and postural control through active self-correction and specific breathing techniques. Core stabilization training targets the enhancement of deep trunk muscle support to improve spinal stability. General exercise or physical activity serves primarily as a non-specific comparator to control for the general effects of physical activity, without specific corrective intent.

Intervention nodes in the network meta-analysis were pre-categorized based on the treatment name reported in the original study, main treatment principles, implementation methods, and clinical interpretability. Schroth training was retained as an independent node, as it is a clearly named and relatively standardized scoliosis-specific training method primarily involving three-dimensional self-correction, postural control, and rotational breathing training. Core stabilization training was defined as training protocols primarily targeting trunk and core muscle activation, neuromuscular control, and spinal stability, without incorporating the complete Schroth-specific corrective system. Scoliosis-specific exercise was used for interventions explicitly described by the original study as SSE, SEAS, Dobomed, or other non-Schroth scoliosis-specific exercise protocols. Traditional exercise or general physical activity was used for interventions not explicitly employing scoliosis-specific corrective principles and primarily serving as general exercise controls. Bracing, TCM-related rehabilitation interventions, and observation/usual care were treated as separate nodes where applicable.

If an intervention in a study comprised multiple treatment components but was implemented and reported by the original study as a holistic protocol, it was classified under the most clinically representative node based on its primary treatment purpose and core components. Node classification was performed independently by two investigators and finalized before data analysis; disagreements were resolved through discussion or by a third investigator.

### 2.6 Risk of bias assessment

Risk of bias was assessed using tools appropriate to the study design. For randomized controlled trials, the Cochrane risk-of-bias tool was used, assessing domains including random sequence generation, allocation concealment, blinding of participants and personnel, blinding of outcome assessment, completeness of outcome data, selective reporting, and other biases.

For prospective non-randomized comparative studies, the ROBINS-I tool was used, assessing domains including confounding bias, selection of participants bias, classification of interventions bias, deviations from intended interventions bias, missing data bias, measurement of outcomes bias, and selective reporting of results bias. Risk of bias results for non-randomized studies were not pooled with those of RCTs but were used separately to evaluate the reliability of supplementary evidence.

Risk of bias assessment was conducted independently by two investigators. Disagreements were resolved through discussion; if consensus could not be reached, a third investigator made the final decision. Risk of bias assessment results were used to interpret the robustness of the primary findings and were not used as a sole criterion for excluding studies.

### 2.7 Data synthesis and statistical analysis

All statistical analyses were performed using Stata 17.0 software. Cobb angle and SRS-22 total score were continuous outcomes. For comparisons with consistent units and clear clinical meaning, mean difference (MD) with 95% confidence interval (CI) was preferentially used as the effect measure; for traditional pairwise meta-analyses where different studies reported forms or effect measure scales varied, standardized mean difference (SMD) with 95% CI was used as a supplementary summary measure. For Cobb angle, negative values indicated a reduction in curve angle after intervention, favoring the experimental intervention.

First, traditional pairwise random-effects meta-analyses were performed for interventions with sufficient direct comparison data, and statistical heterogeneity was evaluated using the I^2^ statistic. Subsequently, a primary random-effects network meta-analysis model was constructed based on RCTs, synthesizing direct and indirect evidence within the same framework to estimate the relative effects of different non-surgical interventions. Prospective non-randomized comparative studies were not included in the effect estimation of the primary network meta-analysis but served as supplementary evidence for descriptive comparisons and robustness interpretation.

Network plots were used to visualize intervention nodes and their direct comparison relationships. Node size was visualized based on the number of direct comparisons involving that intervention in the RCT evidence network, and line thickness reflected the number of studies for the corresponding direct comparison. Relative treatment effects were presented as forest plots or league tables. Treatment ranking was described using the surface under the cumulative ranking curve (SUCRA) and ranking probabilities. SUCRA was used only as a supportive probabilistic summary measure and not as an independent basis for judging the definitive superiority of any intervention; all ranking results were interpreted in conjunction with effect estimates, confidence intervals, between-study heterogeneity, risk of bias, network sparsity, and clinical plausibility.

### 2.8 Assessment of transitivity, consistency, and small-study effects

Before conducting the network meta-analysis, the plausibility of the transitivity assumption was assessed descriptively from clinical and methodological perspectives. Potential effect modifiers across different treatment comparisons were compared, focusing on age, baseline Cobb angle, skeletal maturity indicators, curve severity, intervention duration, follow-up duration, co-interventions, and treatment setting. Due to incomplete reporting of these variables in some original studies, the transitivity assessment was primarily descriptive.

Consistency between direct and indirect evidence was evaluated where the network structure permitted. If certain comparisons could not undergo stable consistency testing due to network sparsity or insufficient closed loops, this was explicitly stated in the result interpretation, and the certainty of interpretation for related indirect comparisons and ranking results was reduced. For outcomes with a limited number of included studies, particularly SRS-22, the overall results were treated as exploratory findings.

Publication bias and small-study effects were explored using funnel plots and Egger's regression test. Given the limited number of included studies, sparse network structure, and presence of between-study heterogeneity, the assessment of small-study effects and publication bias was considered exploratory and not used as a sole basis for determining evidence certainty.

### 2.9 Supplementary analysis and robustness interpretation

To assess the impact of study design differences on result interpretation, prospective non-randomized comparative studies were described separately and used for robustness interpretation as supplementary evidence. The primary conclusions were based on the results of the RCT-based network meta-analysis; non-randomized comparative studies were used only to observe whether the direction of their results aligned with the trend of the primary analysis and to discuss the external applicability and methodological limitations of the existing evidence. For outcomes with sparse evidence, such as SRS-22, over-interpretation of rankings was avoided, and they were presented only as exploratory evidence for patient-reported outcomes.

## 3 Results

### 3.1 Literature search and screening results

**Figure 1:**
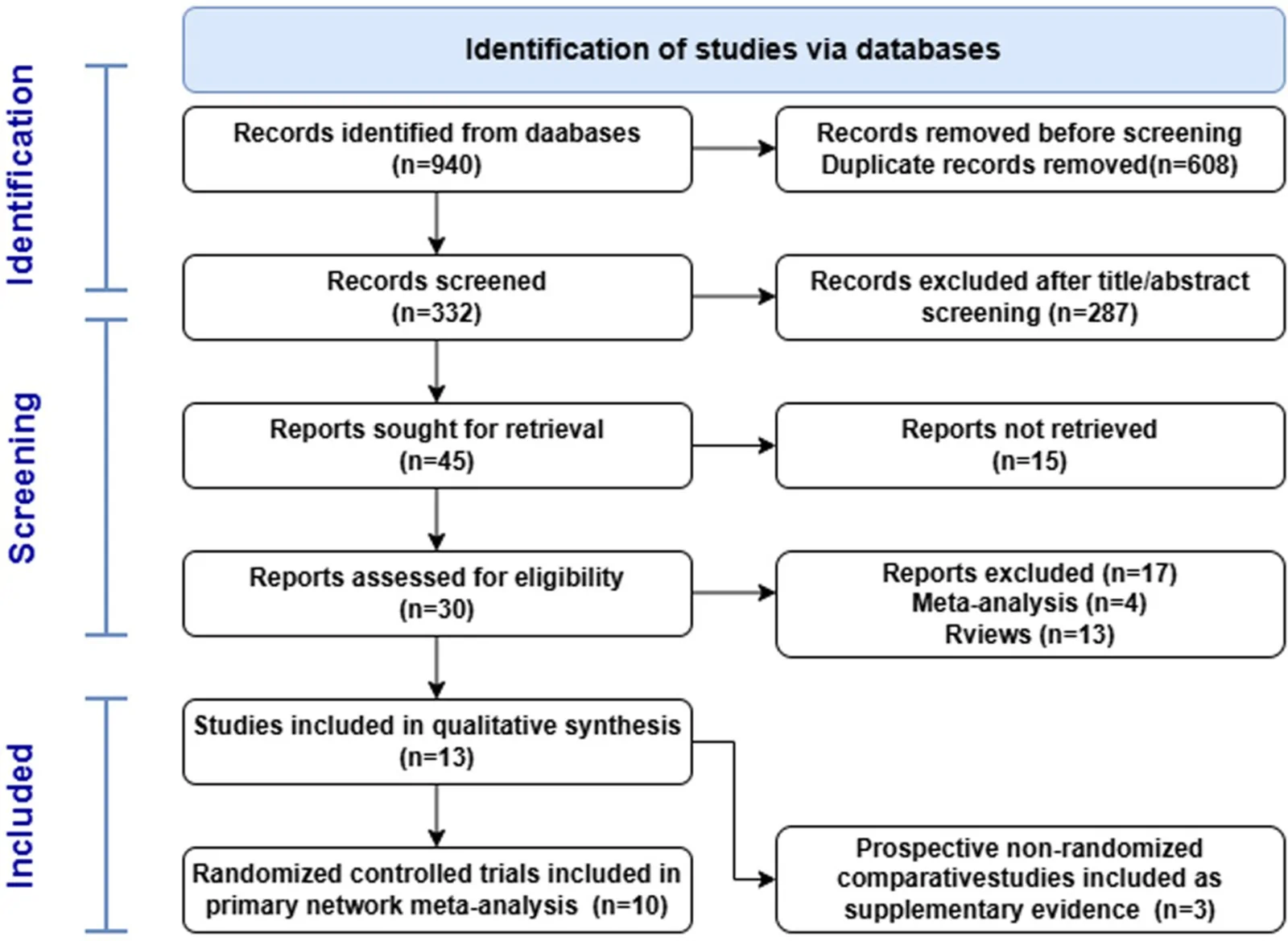
PRISMA flow diagram.

The database search yielded 940 records. After removing 608 duplicate records, 332 records entered title and abstract screening. Following screening, 287 records were excluded, and 45 full-text articles were sought for retrieval. Of these, 15 articles could not be retrieved, and 30 full-text articles underwent eligibility assessment. After full-text assessment, 17 articles were excluded, including 4 meta-analyses and 13 reviews. Finally, 13 comparative intervention studies were included, of which 10 randomized controlled trials were included in the primary network meta-analysis, and 3 prospective non-randomized comparative studies served as supplementary evidence. The study selection flow diagram is presented in Figure 1.

### 3.2 Baseline characteristics of included studies

This systematic review included a total of 13 comparative intervention studies involving 709 AIS participants. Among these, 10 randomized controlled trials included 487 participants, forming the evidence base for the primary network meta-analysis; 3 prospective non-randomized comparative studies included 222 participants and served as supplementary evidence for description and interpretation. The included studies originated from 6 countries: 4 from China, 5 from Turkey, 1 from Canada, 1 from the United Kingdom, 1 from Italy, and 1 from Sweden.

The non-surgical interventions evaluated in the included studies included Schroth training, three-dimensional exercise therapy, scoliosis-specific exercises, core stabilization training, traditional or general physical activity, bracing, observation or usual care, and TCM-related rehabilitation interventions. According to the pre-specified node classification rules, the primary network meta-analysis involved 8 types of intervention nodes. All 13 studies reported Cobb angle outcomes, of which 5 studies also reported SRS-22 total scores. The intervention durations and follow-up periods of the included studies varied considerably, ranging from 10 weeks to 49 months overall. RCTs were predominantly short-term evaluations, with follow-up windows mainly concentrated between 10 weeks and 6 months; a few studies followed up for 12 months or longer. Baseline characteristics, interventions, follow-up durations, and outcome measures of the included studies are presented in Table 1.

**Table 1:** Baseline and transitivity-related characteristics of included studies (as reported by the original studies).

Included research	Type of study	Country	Sample size (E/C)	Age(years old) (as reported, E/C)	Baseline Cobb angle (E/C)	Risser sign/menarche (if reported)	Intervention		Duration of intervention	Follow-up/analyzed time point	Outcome measures
							experiment	control			
Kuru 2016 (9)	Randomized controlled trial (parallel-group)	Turkey	30 (15/15)	12.9 (1.4)/13.1 (1.7)	33.4 ± 8.9/30.3 ± 6.6	Risser sign 1.5 ± 1.3/1.0 ± 1.2; menarche NR	3D exercise	Observation	6 months of treatment	Baseline, 6, 12, and 24 weeks; Cobb angle analyzed at baseline and 24 weeks	Cobb angle
Kocaman 2021 (10)	Randomized controlled trial (single-blind; parallel-group; 1:1)	Turkey	28 (14/14)	14.07 (2.37)/14.21 (2.19)	Thoracic 17.64 ± 4.01/17.29 ± 3.45; Lumbar 15.80 ± 3.42/15.17 ± 4.02	Risser sign 1.64 ± 1.34/1.78 ± 1.19; menarche NR	Schroth exercise	Core exercise	10 weeks of treatment	Baseline and post-treatment after 10 weeks	Cobb angle; SRS-22
Schreiber 2016 (11)	Randomized controlled trial (phase II; assessor/statistician blinded; 1:1)	Canada	50 (25/25)	13.5 (12.7–14.2)/13.3 (12.7–13.9)	Largest curve 29.1/27.9	Risser sign 1.76/1.44; menarche NR	Schroth exercise	Observation or bracing with SRS recommended dosage	6 months of treatment	Baseline and 6-month follow-up	Cobb angle; SRS-22
Gur 2017 (12)	Randomized controlled trial (parallel-group)	Turkey	25 (12/13)	14.2 (1.8)/14 (1.6)	Thoracic 35.0 ± 11.82/31.42 ± 6.97; Lumbar 29.0 ± 8.35/34.33 ± 9.20; Total 56.75 ± 25.70/60.69 ± 17.75.	Risser grade (E/C): 2.0 ± 0.6/2.0 ± 0.6; Menarche status: NR	Core exercise	Traditional exercise	10 weeks of treatment	Pre-treatment and post-treatment after 10 weeks	Cobb angle; SRS-22
Liu 2020 (13)	Non-randomized controlled study (prospective controlled cohort)	China	99 (53/46)	9.7 (0.8)/13.6 (0.8)	NR	Risser NR; menarche NR (inclusion criteria: Cobb angle 10°–25°, Risser grade 0–3)	3D exercise	Traditional exercise	49 months of treatment	Baseline; monitored every 3–4 months; follow-up >1 year	Cobb angle
Charalampidis 2024 (14)	Randomized controlled trial (parallel-group)	Sweden	90 (45/45)	12.6 (1.4)/12.6 (1.5)	31 (4)/31 (4)	Risser grade distribution reported; menarche NR	SSE	Physical exercise	6 months of treatment	Baseline and every 6-month follow-up until skeletal maturity; followed for 2 years after the primary outcome	Cobb angle
Williams 2015 (15)	Pilot randomized controlled trial (feasibility RCT)	UK	50 (25/25)	10–16	Treatment A 34.3 ± 10.1; Treatment B 33.8 ± 10.1	Risser classification distribution reported; menarche NR	SSE	Physical exercise	6 months of treatment	Baseline and 6 months after randomisation	Cobb angle
Fong 2015 (16)	Non-randomized controlled study (comprehensive cohort design)	China	49 (18/31)	12.4 (0.9)/12.7 (1.2)	26.4 ± 4.1/24.9 ± 3.0	Risser 0–2 in inclusion criteria; baseline group-specific values NR; menarche NR	Brace	Observation	12 months treatment; mean follow-up 168 weeks	Every 4 months for a minimum of 12 months; mean follow-up 168 weeks overall; curve progression assessed during follow-up	Cobb angle; SRS-22
Negrini 2008 (17)	Non-randomized controlled study (prospective controlled cohort)	Italy	74 (35/39)	12.7 (2.2)/12.1 (2.1)	NR; overall cohort mean 15° (SD 6°)	Baseline group-specific values NR; pre-menarchal status and Risser-based criteria described; Risser >3 excluded	Brace	Physical exercise	12 months of treatment	Clinical follow-up every 6 months; radiographic assessment after 12 months of treatment	Cobb angle
Manzak 2024 (18)	Randomized controlled trial (evaluator-blinded)	Turkey	31 (16/15)	10–18	NR	Risser classification evaluated, but baseline group-specific values not verified; menarche NR	Pilates	SSE	12 weeks of treatment	Baseline and end of study after 12 weeks	Cobb angle
Büyükturan 2024 (19)	Randomized controlled trial (assessor/statistician blinded)	Turkey	31 (15/16)	14.0 (1.9)/14.2 (2.0)	Thoracic (CA-T) 22.8 ± 3.1/20.4 ± 2.2; Lumbar (CA-L) 18.3 ± 3.7/17.1 ± 1.9	Risser sign distribution reported (SG: 0/1/2/3 = 2/3/6/4; LG: 2/3/6/5); menarche NR	Schroth exercise	Lyon exercise	6 months of treatment	Baseline and post-treatment after 6 months	Cobb angle; SRS-22
Gao 2019 (20)	Randomized controlled trial (parallel-group)	China	45 (22/23)	12.14 (1.32)/ 12.22 (1.35)	29.13 ± 4.32/28.64 ± 3.91	Risser 0–2 and premenarchal or <1 year postmenarchal in inclusion criteria; baseline group-specific values NR	Schroth exercise	Brace	6 months of treatment	Baseline, 1-month, and 6-month follow-up; Cobb angle analyzed at baseline and 6 months	Cobb angle
Wei 2015 (21)	Randomized controlled trial (parallel-group)	China	107 (58/49)	9.1 (0.4)/8.9 (0.6)	30.4 ± 3.7/31.5 ± 3.2	Risser 0–2 and premenarche or within 1 year of menarche onset in inclusion criteria; baseline group-specific values NR	Daoyin,Tuina, and acupotomology therapies	Brace	12 months treatment; 12 months follow-up	Baseline; Cobb angle analyzed after 12 and 24 months of treatment	Cobb angle

Footnotes: E/C, experimental group/control group; SRS-22, Scoliosis Research Society-22 questionnaire; SSE, scoliosis-specific exercises; NR, not reported in the original study. Baseline and transitivity-related characteristics were extracted and summarized as reported by the original studies. Skeletal maturity indicators, co-interventions, and analyzed follow-up time points were incompletely reported across several studies.

RCT, randomized controlled trial; NRSI, non-randomized study of interventions.

### 3.3 Risk of bias assessment results

**Figure 2:**
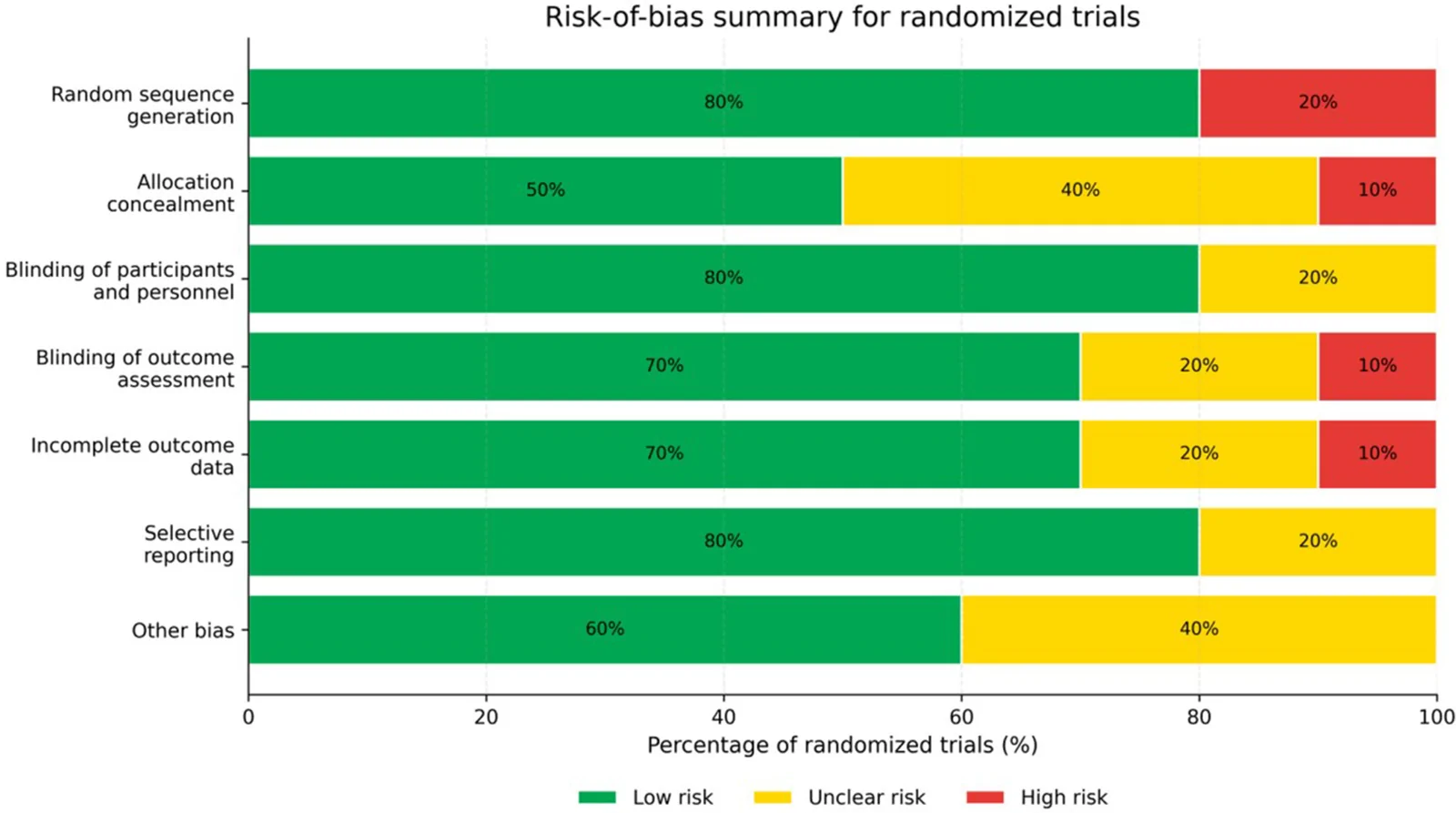
Risk of bias summary for RCTs—bar chart.

**Figure 3:**
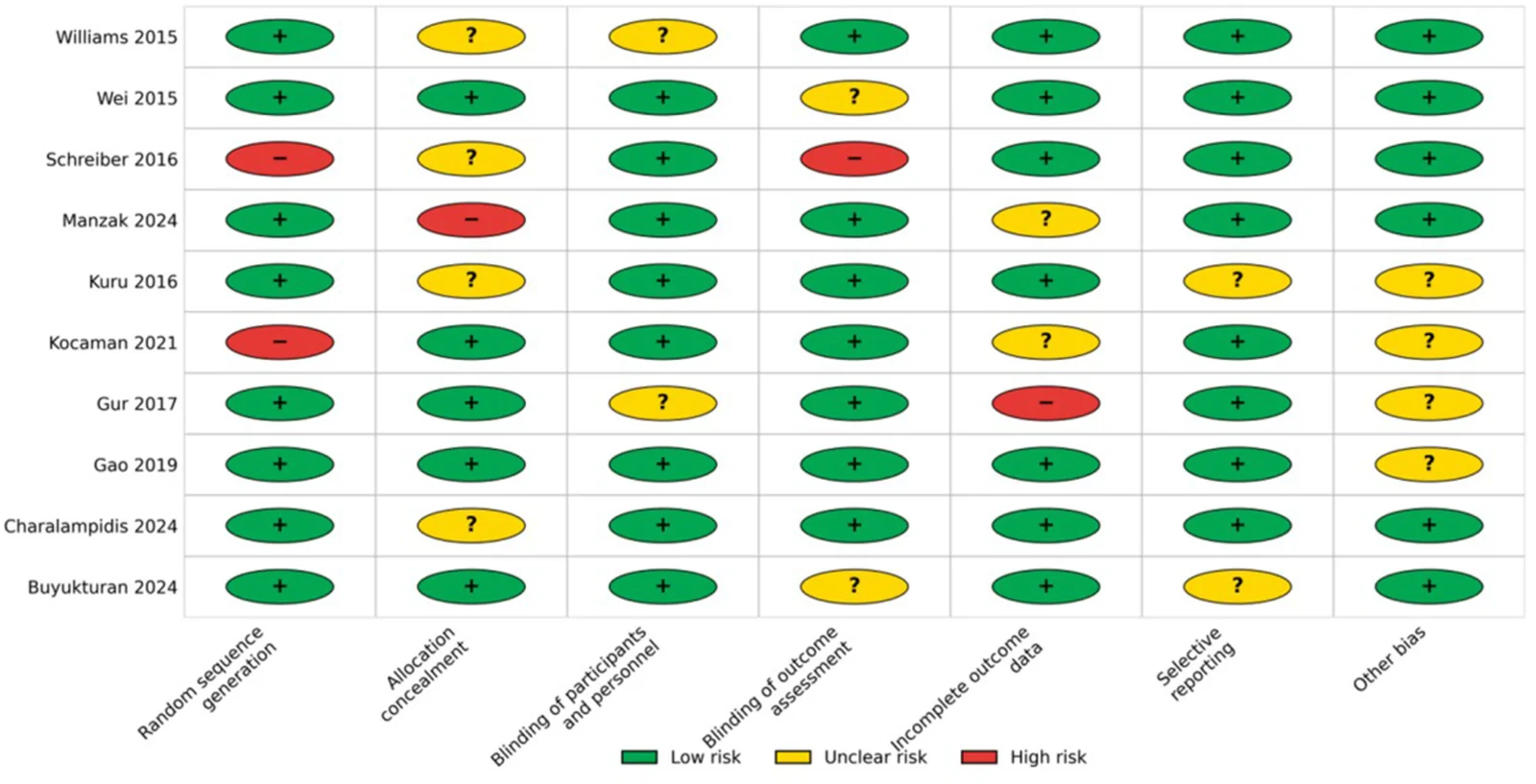
Risk of bias traffic light plot for RCTs.

The 10 randomized controlled trials were assessed using the Cochrane risk-of-bias tool. Overall, most RCTs were judged as having low risk of bias or some concerns in certain domains. Sources of bias mainly originated from inadequate reporting of the randomization process, incomplete information on allocation concealment, difficulty blinding participants or personnel during intervention implementation, and limited information on outcome measurement or reporting in some studies. A summary of the risk of bias for the 10 RCTs is presented in Figure 2, and the study-level risk of bias judgments are presented in Figure 3.

The 3 prospective non-randomized comparative studies were assessed separately using the ROBINS-I tool. Compared to RCTs, the non-randomized comparative studies had potential risks of bias related to confounding, selection of participants, and intervention assignment. Therefore, these studies were not included in the effect estimation of the primary network meta-analysis but served only as supplementary evidence for interpreting the directionality and external applicability of the primary findings.

### 3.4 Network evidence structure

The primary network meta-analysis was constructed based on the 10 randomized controlled trials. The network evidence plot for the Cobb angle outcome showed that the included studies formed an evidence network consisting of various non-surgical intervention nodes. Each node represented a pre-specified intervention category, node size reflected the number of direct comparisons involving that intervention in the RCT evidence network, lines between nodes indicated the presence of direct comparisons, and line thickness reflected the number of studies for that comparison. Overall, general exercise/usual care, Schroth training, scoliosis-specific exercises, and traditional exercise were relatively more connected nodes in the Cobb angle evidence network, but the number of direct comparison studies between most nodes was limited, indicating that the overall evidence network was relatively sparse.

**Figure 4:**
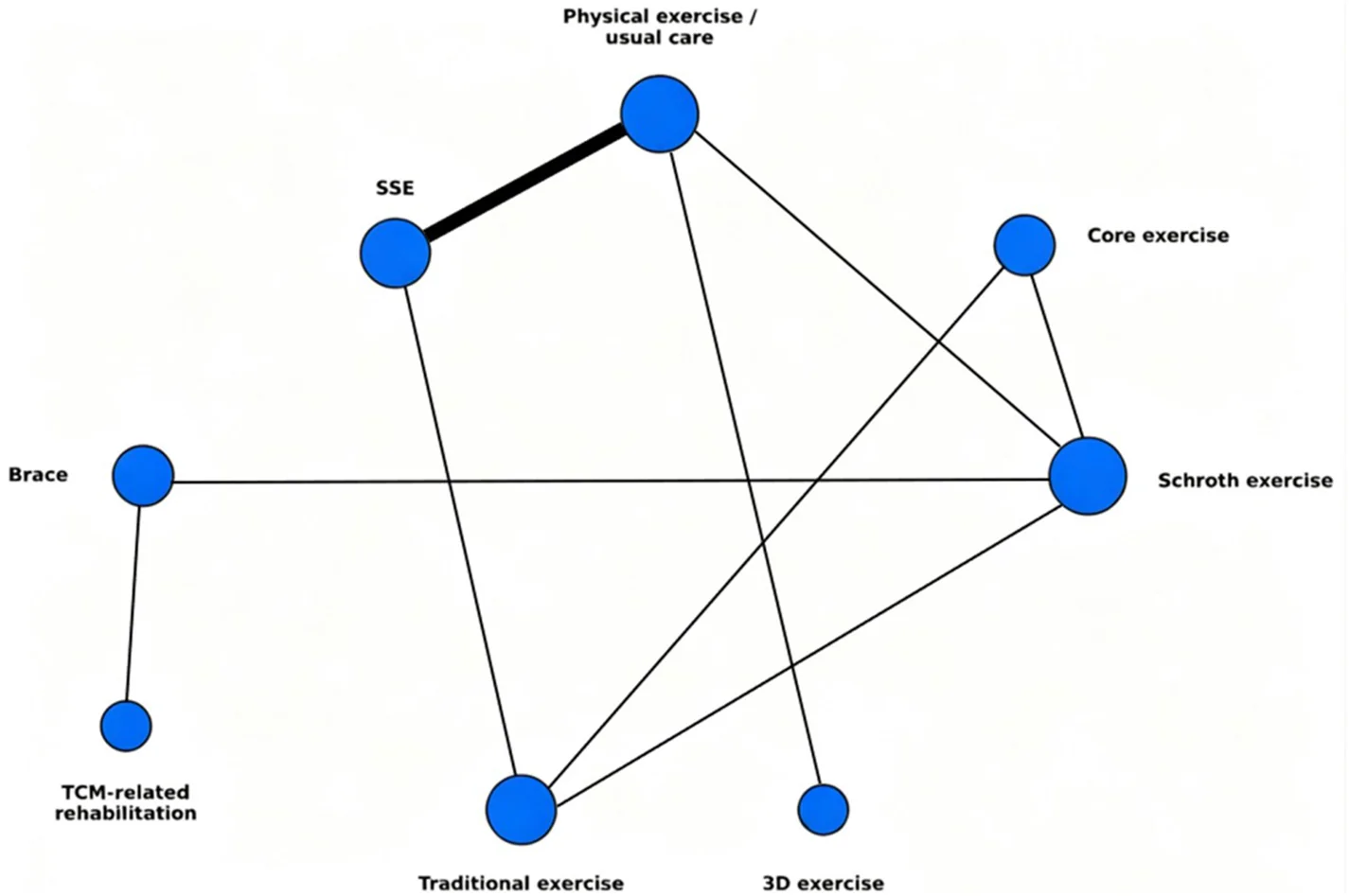
Network plot for Cobb angle.

**Figure 5:**
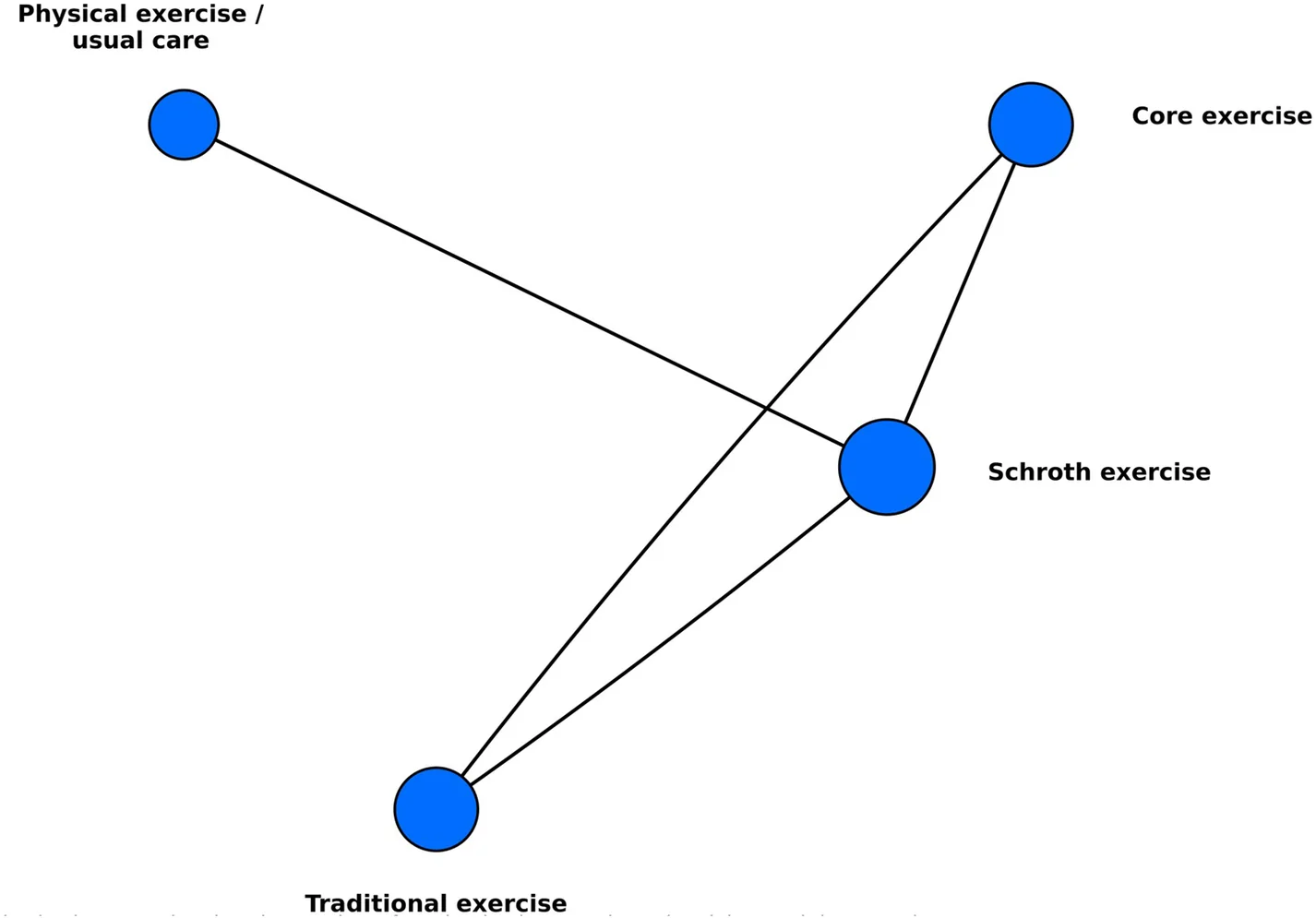
Network plot for SRS-22 total score.

The network evidence for the SRS-22 total score outcome was considerably sparser than for Cobb angle. Only some RCTs reported SRS-22 total scores, resulting in a much sparser network structure for this outcome; thus, related comparisons and ranking results should be considered exploratory. The network evidence structures for Cobb angle and SRS-22 outcomes are presented in Figures 4, 5, respectively.

### 3.5 Network meta-analysis results for Cobb angle outcome

The primary network meta-analysis evaluated the relative effects of different non-surgical interventions on Cobb angle based on RCTs. Direct comparison effect estimates from RCTs contributing to the Cobb angle evidence network are presented in Table 2. Overall, some direct comparison results suggested that Schroth-related therapies, scoliosis-specific exercises, core stabilization training, and some exercise-based interventions may be beneficial for short-term Cobb angle control. However, the 95% CIs for some comparisons were wide or crossed the line of no effect, and some effect estimates originated from single studies or complex intervention nodes. Therefore, these direct comparison effect estimates should not be interpreted alone as evidence of definitive superiority of any intervention but should be interpreted jointly with the network evidence structure, SUCRA rankings, between-study heterogeneity, risk of bias, and clinical plausibility.

**Table 2:** Direct comparative effect estimates for Cobb angle among randomized controlled trials contributing to the evidence network.

Study	Direct comparison	Network node classification	SMD (95% CI)	Direction of effect
Wei 2015 (21)	Traditional Chinese medicine-related rehabilitation vs. brace treatment	TCM-related rehabilitation vs. Brace	0.79 (0.39, 1.18)	Favors brace treatment
Kocaman 2021 (10)	Schroth exercises vs. core stabilization exercises	Schroth exercise vs. Core exercise	−8.08 (−10.40, −5.76)	Favors Schroth exercise
Schreiber 2016 (11)	Schroth exercises plus standard care vs. standard care	Schroth exercise vs. Physical exercise/usual care	−0.29 (−0.85, 0.26)	Uncertain
Gur 2017 (12)	Core stabilization exercises vs. traditional rehabilitation	Core exercise vs. Traditional exercise	−0.89 (−1.71, −0.06)	Favors core exercise
Kuru 2016 (9)	Three-dimensional Schroth exercises vs. control/usual care	3D exercise vs. Physical exercise/usual care	−1.59 (−2.41, −0.76)	Favors 3D exercise
Charalampidis 2024 (14)	Scoliosis-specific exercise vs. physical exercise	SSE vs. Physical exercise/usual care	−0.03 (−0.44, 0.39)	Uncertain
Williams 2015 (15)	Scoliosis-specific exercise vs. standard care	SSE vs. Physical exercise/usual care	−1.18 (−1.78, −0.58)	Favors SSE
Manzak 2024 (18)	Pilates-based exercise with hybrid telerehabilitation vs. scoliosis-specific exercise	Traditional exercise vs. SSE	−2.53 (−3.47, −1.59)	Favors Pilates-based exercise
Büyükturan 2024 (19)	Schroth exercises vs. Lyon exercises	Schroth exercise vs. Traditional exercise	−0.59 (−1.31, 0.13)	Uncertain
Gao 2019 (20)	Schroth exercises vs. brace treatment	Schroth exercise vs. Brace	0.75 (0.15, 1.36)	Favors brace treatment; interpret cautiously

Values are study-level standardized mean differences (SMDs) with 95% confidence intervals (CIs) for Cobb angle. Negative values indicate a greater reduction in Cobb angle in the first-listed intervention compared with the comparator, whereas positive values indicate a direction favoring the comparator. This table summarizes direct comparative effect estimates from randomized controlled trials contributing to the Cobb angle evidence network and does not represent a full network league table. For complex or named exercise interventions, node classification was based on the main therapeutic component and the prespecified intervention taxonomy used in this review. These estimates should be interpreted together with the network structure, SUCRA ranking, heterogeneity, risk of bias, and clinical plausibility.

**Figure 6:**
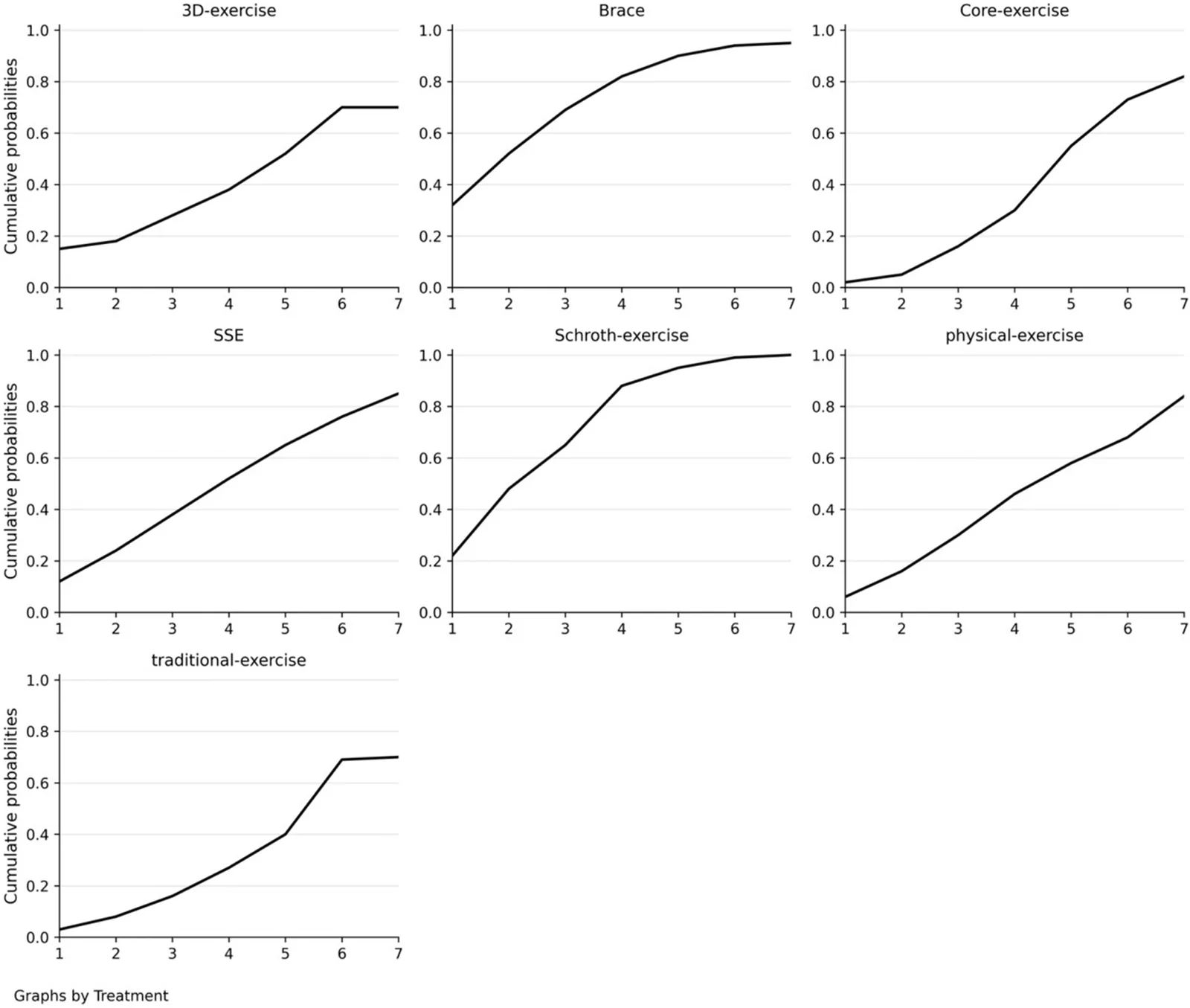
Cumulative ranking probability plots for Cobb angle.

SUCRA ranking results suggested that bracing and Schroth-related therapies may have relatively better performance for short-term Cobb angle control. However, SUCRA only reflects probabilistic ranking and cannot substitute for effect estimates and their uncertainty. Therefore, ranking results are presented as supportive descriptive indicators and interpreted in conjunction with confidence intervals, between-study heterogeneity, risk of bias, and clinical plausibility. The cumulative probability ranking plot for the Cobb angle outcome is presented in Figure 6.

### 3.6 Traditional pairwise meta-analysis results for Cobb angle outcome

**Figure 7:**
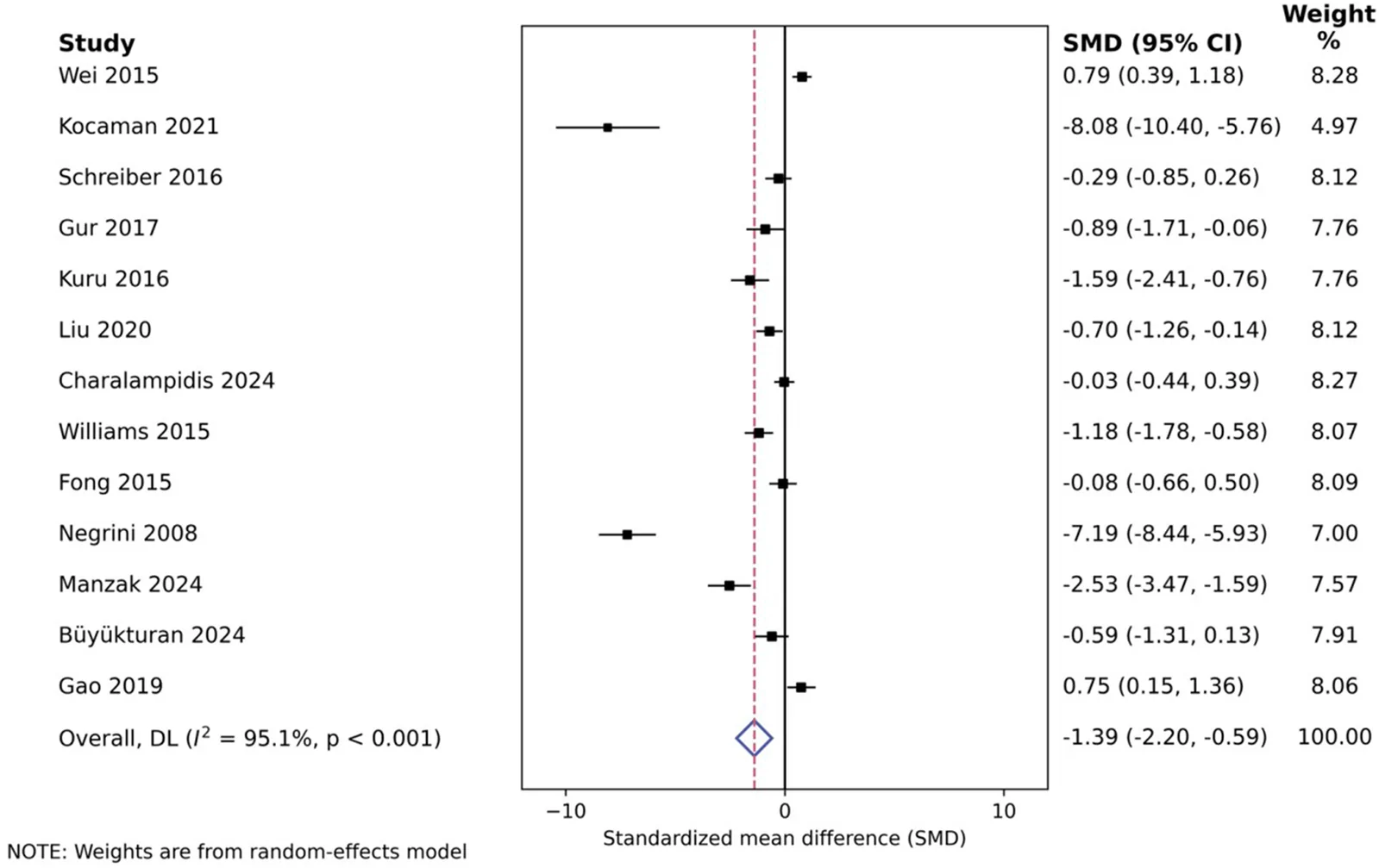
Forest plot for pairwise meta-analysis of Cobb angle.

To complementarily describe the overall characteristics of Cobb angle effect estimates across the 13 comparative intervention studies, a traditional pairwise random-effects meta-analysis was further conducted. The results showed considerable variability in study-level effect estimates for Cobb angle. The pooled standardized mean difference was −1.39 (95% CI: −2.20 to −0.59), suggesting an overall direction favoring non-surgical interventions for potentially improving Cobb angle. However, statistical heterogeneity was substantial (I^2^ = 95.1%, *P* < 0.001), indicating significant clinical and methodological differences across studies. The study-level SMD ranged from −8.08–0.79, further suggesting that intervention type, treatment intensity, follow-up duration, participant baseline characteristics, and outcome extraction methods may importantly influence results. Therefore, this pooled effect estimate should be interpreted cautiously as an overall descriptive result and not as evidence of consistent efficacy across all non-surgical interventions. The forest plot for the traditional pairwise random-effects meta-analysis of Cobb angle is presented in Figure 7.

### 3.7 Network meta-analysis results for SRS-22 outcome

**Figure 8:**
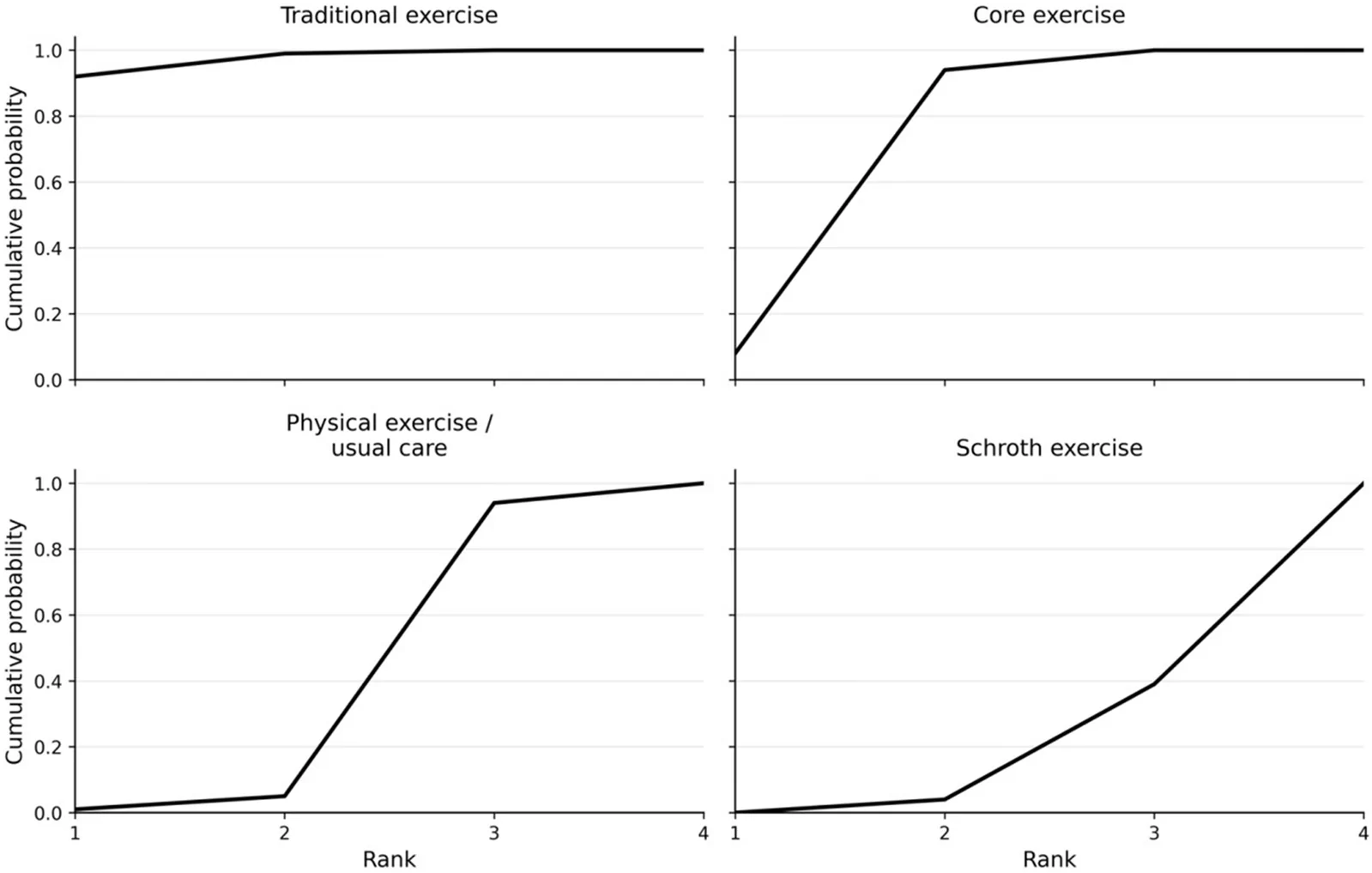
Cumulative ranking plot for SRS-22 total score.

The SRS-22 total score, as a patient-reported outcome, was used to evaluate the impact of non-surgical interventions on health-related quality of life. Compared to Cobb angle, available evidence for the SRS-22 outcome was considerably less. A total of 5 studies reported SRS-22 total scores, of which 4 RCTs were included in the RCT-based network analysis, and 1 prospective non-randomized comparative study served only as supplementary evidence. Due to the limited sample size, sparse network structure, and small number of direct comparisons, the SRS-22-related ranking results can only be considered exploratory evidence for patient-reported outcomes and are insufficient to support definitive judgments of superiority among different interventions for improving quality of life. Existing evidence only suggests that some non-surgical interventions may have potential benefits for patient-reported outcomes, but further validation with adequately powered RCTs, consistent outcome reporting, and longer follow-up durations is needed. The ranking plot for SRS-22 total score is presented in Figure 8.

### 3.8 Assessment of small-study effects and publication bias

**Figure 9:**
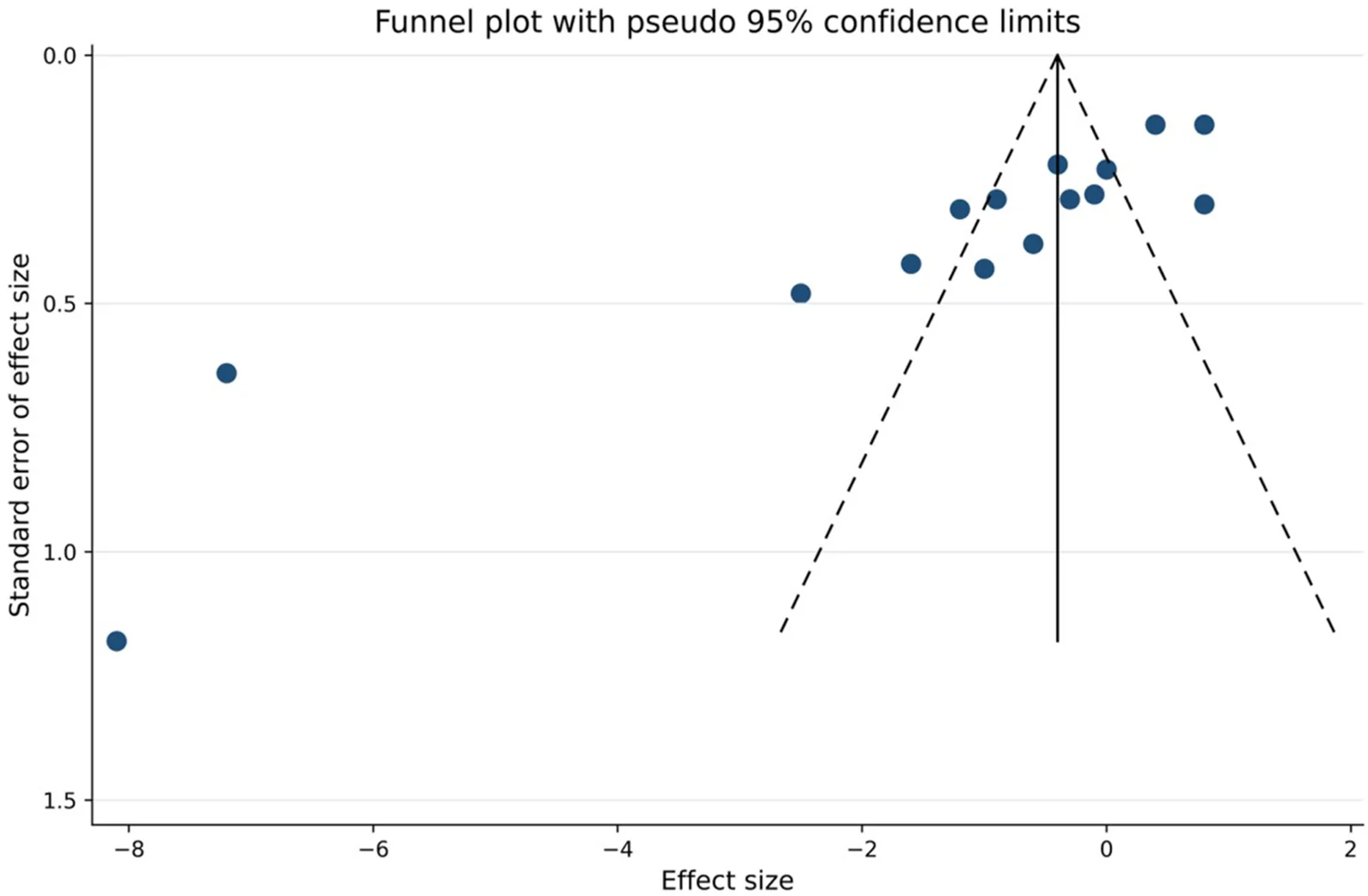
Funnel plot for direct comparisons).

**Figure 10:**
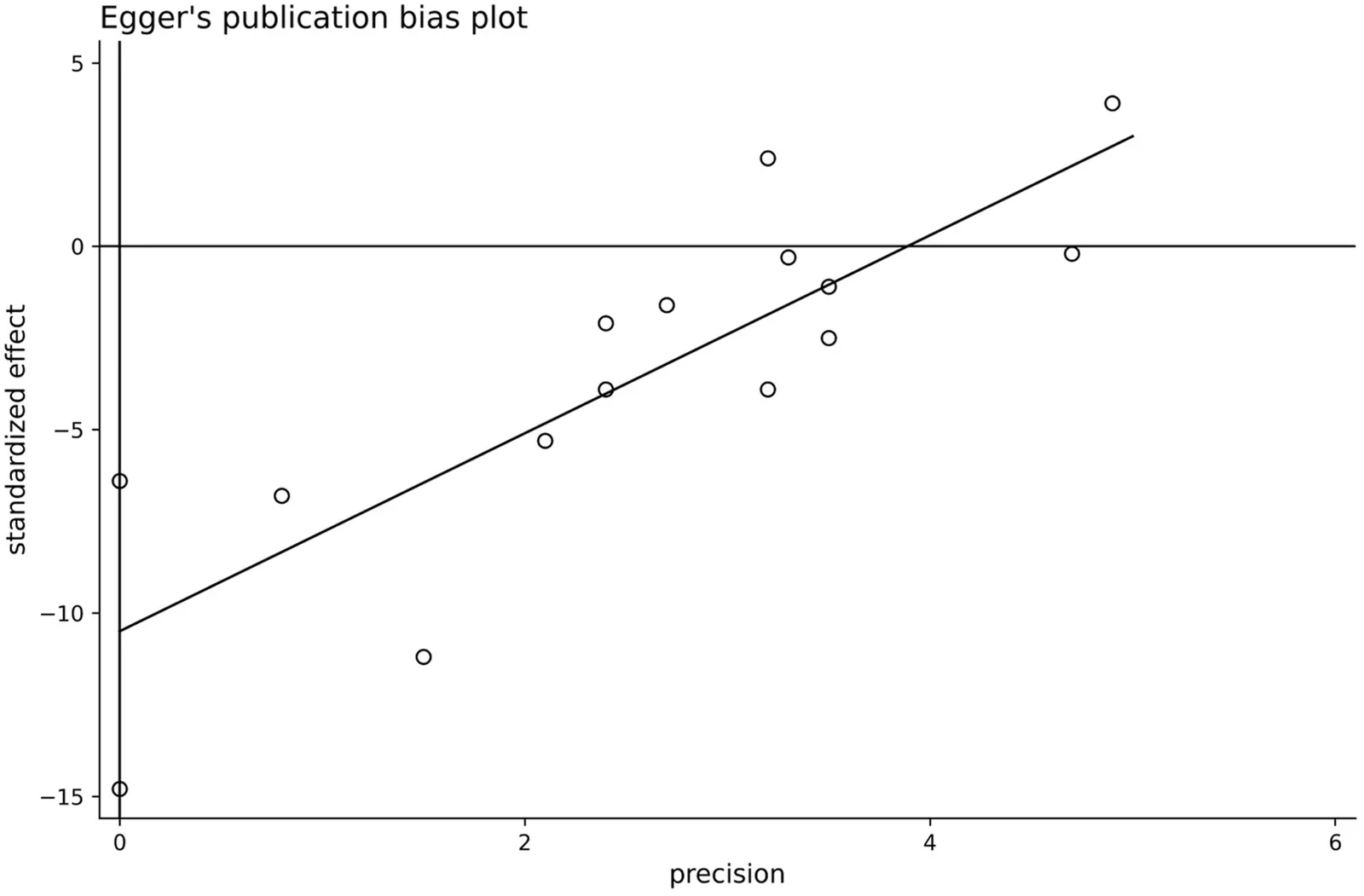
Egger's regression plot.

This study explored publication bias and small-study effects for the Cobb angle outcome using funnel plots and Egger's regression test. Visual inspection of the funnel plot suggested possible asymmetry, and the Egger's regression plot also indicated potential small-study effects. However, given the limited number of included studies, sparse network structure, and significant between-study heterogeneity, the results of the publication bias and small-study effects assessment are only exploratory and should not be used as the sole basis for determining evidence certainty. The relevant plots are presented in Figures 9, 10.

### 3.9 Supplementary evidence from prospective Non-randomized comparative studies

The three prospective non-randomized comparative studies included Liu 2020, Fong 2015, and Negrini 2008. These studies evaluated non-surgical interventions such as three-dimensional exercise therapy, bracing, or specific exercises using prospective controlled cohort designs, comprehensive cohort designs, or prospective observational controlled studies. Because these studies were not standard RCTs and had potential risks of confounding, selection bias, and intervention allocation bias, they were not included in the effect estimation of the primary network meta-analysis.

From the perspective of supplementary evidence, these non-randomized comparative studies provide some reference for long-term follow-up and real-world application of AIS non-surgical treatment, but their internal validity is lower than that of RCTs. Their results were primarily used to observe whether the direction of effects of different interventions was generally consistent with the primary RCT evidence and to support discussion of external applicability and methodological limitations. Results based on these studies were not used as a basis for the primary conclusions of this study.

## 4 Discussion

This systematic review and network meta-analysis compared the effects of commonly used non-surgical interventions for AIS on Cobb angle and SRS-22 outcomes. Overall, the available evidence suggests that some non-surgical interventions may help improve Cobb angle in AIS patients, with bracing and Schroth-related therapies showing a trend toward relative superiority for short-term Cobb angle control. However, this conclusion should be interpreted cautiously against the backdrop of a sparse evidence network, significant between-study heterogeneity, imprecise estimates for some comparisons, and potential small-study effects. Compared to Cobb angle, evidence related to SRS-22 is markedly insufficient, the network structure is much sparser, and the associated ranking results should only be considered exploratory findings, not as evidence of definitive superiority of any intervention for improving quality of life (22).

Recent network meta-analyses in other clinical fields based on RCTs provide important methodological references for interpreting the results of this study. For example, Tzang et al. (6), when evaluating immune checkpoint inhibitors for colorectal cancer, employed network evidence including only RCTs and emphasized interpreting comparative results based on effect estimates, confidence intervals, and consistency assessments, rather than relying solely on ranking probabilities. Similarly, Tsai et al. (7), in a network meta-analysis of exercise interventions for primary dysmenorrhea, reduced the impact of excessive heterogeneity on result interpretation by limiting comparisons with excessive clinical heterogeneity and classifying intervention nodes based on actual exercise content rather than original study labels. These methodological experiences align with the principles adopted in this study, namely that SUCRA rankings serve only as supportive probabilistic indicators and should not be interpreted alone as demonstrating definitive superiority of any treatment.

The primary finding of this study is that bracing and Schroth-related therapies showed higher ranking trends for Cobb angle control. This result is generally consistent with current clinical knowledge and guideline recommendations for AIS conservative treatment (22–25). Bracing is an important strategy for controlling curve progression in patients with mild-to-moderate AIS who still have growth potential, primarily by applying continuous external corrective forces to reduce the risk of curve progression. However, the real-world efficacy of bracing is closely related to patient compliance, and brace wearing may impose psychological burden, physical activity limitations, and socioeconomic stress, thereby affecting actual wearing time and treatment effectiveness (26). Furthermore, even among patients reporting good compliance, some may still progress to the surgical threshold, suggesting significant individual variability in response to bracing, possibly influenced by factors such as baseline Cobb angle, skeletal maturity, curve type, and treatment intensity (27). During long-term follow-up and brace adjustments, patients typically require repeat radiographic examinations, raising concerns about cumulative ionizing radiation exposure (28, 29). Therefore, the ranking advantage of bracing should not be simplistically interpreted as being suitable for all AIS patients but should be assessed individually based on patient progression risk, skeletal maturity, compliance, psychological acceptance, and follow-up accessibility (26, 30). Moreover, since the primary goal of AIS bracing is typically to control curve progression until or near skeletal maturity, and the RCTs included in this study were predominantly short-term evaluations, the current results are more suitable as evidence for short-term Cobb angle control trends and are insufficient to directly demonstrate long-term, stable, generalizable benefits of bracing for all AIS patients.

Furthermore, clinical guidelines recommend that physical therapy should complement brace wear rather than serve as a standalone alternative in patients with moderate curves (22, 24). However, the evidence network in our analysis was too sparse to evaluate combination therapies (bracing plus exercise) as independent nodes; most included studies assessed either bracing alone or exercise alone, with very limited head-to-head comparisons of combined vs. single-modality approaches. This represents an important evidence gap that should be addressed in future trials, particularly for patients with curves between 20° and 40° where combination therapy is most commonly prescribed in clinical practice.

Schroth training also showed a trend toward relative superiority for Cobb angle control, with potential mechanisms related to three-dimensional self-correction, postural control, rotational breathing training, and neuromuscular re-education (31, 32). Compared to general exercise, Schroth training emphasizes active correction specific to the individual's curve type and enhances patients’ perception of trunk alignment and postural control through repeated training. This specific training mode may help improve trunk asymmetry, trunk rotation, and spinal posture, thereby potentially influencing Cobb angle and subjective patient function (23, 31). However, the actual effectiveness of Schroth training is highly dependent on therapist experience, patient comprehension, training frequency, home exercise performance, and treatment fidelity. Previous research also suggests variability in implementation details, treatment dose, supervision intensity, and study reporting quality across different Schroth protocols (24, 33, 34). These differences may be important reasons for the unstable effect estimates observed in this study. Therefore, the ranking advantage of Schroth-related therapies should be interpreted as a promising comparative signal, not a definitive efficacy conclusion. Future research needs to further standardize therapist training, treatment dose, home exercise compliance documentation, and treatment fidelity assessment to improve the reproducibility and interpretability of such studies (34–36). Furthermore, even within the named intervention “Schroth,” substantial variability exists across different schools and centers in terms of specific exercise content, dose, supervision, and home-program components. The original Schroth method emphasized non-gravitational opening of the apical concavity combined with rotational breathing directed into the concavity, but subsequent adaptations have introduced significant modifications. Similarly, SEAS is better characterized as a therapeutic approach rather than a single fixed protocol, emphasizing active self-correction and task-oriented exercises rather than a rigid sequence of movements (24). These within-node variations likely contribute to the high between-study heterogeneity observed in our analysis. Regarding potential mechanisms, some authors have linked mild curvatures to delayed maturation of the extrapyramidal system and suggested that TCM-related interventions may influence proprioceptive afferents (Burwell and Grivas), but these mechanistic hypotheses remain speculative and were not directly tested in our study. The mechanisms underlying Schroth‘s potential effects on spinal alignment remain debated. In contrast to the hypothesis that Schroth training improves sensorimotor integration, Zagalaz-Anula et al. found that patients with AIS who received conservative treatment (including Schroth-based exercises) showed worse perception of verticality compared to those managed by observation alone, suggesting that non-gravitational alignment principles may paradoxically impair verticality perception (44). This counterintuitive finding highlights the need for further research on sensorimotor integration and the potential trade-offs between curve correction and proprioceptive function in AIS rehabilitation. Our study was not designed to evaluate mechanistic hypotheses, but this mechanistic uncertainty should be considered when interpreting the clinical results of Schroth-based interventions.

It is also important to recognize that Cobb angle measurement has an inherent variability of approximately 5° due to differences in patient positioning, end-vertebra selection, and reader interpretation (28, 29). Thus, reported changes of less than 5° should not be interpreted as clinically meaningful improvements, even if statistically significant. In our analysis, many of the pairwise comparisons and SUCRA rankings were based on effect estimates with wide confidence intervals and small numerical differences that may fall within this measurement error range. Readers should therefore focus on the direction and consistency of effects across studies rather than the precise magnitude of point estimates. This measurement uncertainty further supports our cautious interpretation of treatment rankings and reinforces that SUCRA probabilities should not be equated with clinically meaningful superiority.

In this study, exercise-based interventions such as core stabilization training, scoliosis-specific exercises, three-dimensional exercise therapy, and traditional exercise were also compared. The potential value of exercise therapy lies not only in improving radiographic parameters but also in enhancing trunk muscle control, improving postural stability, increasing physical function, and promoting active patient participation in treatment (32, 33). However, the definition of exercise-based interventions in original studies is not entirely consistent. Some studies used structured scoliosis-specific training, others used general physical activity, core training, or culturally specific mind-body exercises; different protocols vary considerably in treatment goals, exercise dose, and corrective specificity (24, 33). Therefore, treating different exercise protocols as homogeneous interventions is inappropriate. This study attempted to distinguish between Schroth training, scoliosis-specific exercises, core stabilization training, and traditional exercise through pre-specified node classification, but due to the limited number of available studies, some nodes were still supported by only a few studies, and the corresponding comparative results should be interpreted cautiously.

The SRS-22, a commonly used health-related quality of life assessment tool for AIS patients, reflects subjective patient benefits in terms of pain, function, mental health, self-image, and treatment satisfaction. This study found that network evidence for SRS-22 was considerably sparser than for Cobb angle, with only a few studies reporting this outcome. Therefore, although some exercise-related interventions showed higher probabilities in SRS-22 rankings, the stability and clinical interpretative value of this finding are limited (22). Improvement in Cobb angle does not necessarily equate to improvement in quality of life; patient-reported outcomes may also be influenced by multiple factors including pain levels, appearance perception, treatment burden, brace compliance, family support, and psychological factors (23, 35). Of particular note, a higher SUCRA value for traditional exercise in SRS-22 rankings may reflect ranking instability in a sparse network rather than robust clinical superiority for improving quality of life. Therefore, future AIS non-surgical treatment research should not rely solely on radiographic parameters but should include SRS-22 or other validated patient-reported outcomes in core outcome sets, evaluated at consistent follow-up time points (22, 24).

This study included TCM-related rehabilitation interventions because methods such as tuina (massage), acupuncture, and daoyin have some basis for use in AIS conservative treatment practice in certain regions and fall within the scope of non-surgical treatment according to this study's scope (37, 38). However, existing evidence is insufficient to support TCM-related rehabilitation interventions as structural treatments replacing bracing or standard scoliosis-specific exercises. TCM-related rehabilitation methods may have adjunctive effects on patients by improving soft tissue tension, pain perception, postural control, and treatment compliance, but their impact on three-dimensional spinal structural deformity and long-term curve progression lacks high-quality evidence support (37, 38). Therefore, this study maintains a cautious interpretation of TCM-related rehabilitation interventions, considering their results more as adjunctive or exploratory evidence rather than definitive efficacy evidence.

From a clinical practice perspective, AIS non-surgical management often involves not a single treatment modality but comprehensive decision-making based on patient age, skeletal maturity, Cobb angle magnitude, progression risk, quality of life, compliance, and family support. Bracing may be more suitable for patients with clear progression risk who are still in the growth period, whereas Schroth training and other scoliosis-specific exercises may have value in improving active postural control, enhancing treatment engagement, and adjunctively supporting bracing (10, 30). Previous research also suggests that multimodal approaches such as bracing combined with Schroth training, Schroth training combined with pelvic rotation correction, Schroth training combined with manual therapy, and bracing combined with acupuncture or TCM comprehensive therapy may have potential clinical utility (39–43). However, this study did not model combined treatments as independent nodes adequately and therefore cannot directly infer the superiority of combined therapy over monotherapy. Future research should target multimodal approaches such as bracing combined with Schroth training, bracing combined with other scoliosis-specific exercises, and exercise combined with adjunctive rehabilitation therapies, conducting well-designed, adequately powered RCTs with sufficient follow-up durations (30, 39–43).

An important methodological issue in this study is the plausibility of the transitivity assumption. Network meta-analysis requires that potential effect modifiers across different treatment comparisons be sufficiently similar to allow valid inference from indirect evidence. However, in AIS non-surgical treatment studies, patient baseline Cobb angle, skeletal maturity, Risser sign, menarcheal status, intervention duration, follow-up duration, and co-interventions may all influence treatment effects (22, 24, 27). A particular concern is that different non-surgical interventions are not always used in clinically overlapping patient populations: exercise-based interventions are more commonly applied to milder curves (<25°), whereas bracing is more frequently prescribed for moderate curves (20°–40°) with documented progression risk. In clinical practice, bracing is typically more commonly used for patients with moderate curves who still have growth potential and clear progression risk, while exercise therapy or adjunctive rehabilitation interventions may be applied across a broader range of curve severities and treatment scenarios. Because some original studies incompletely reported these key variables, transitivity could only be assessed descriptively and could not be fully validated through formal statistical methods. Therefore, indirect comparisons involving brace vs. non-brace interventions, specific exercise vs. general exercise, and short-term vs. long-term follow-up should all be interpreted cautiously.

This study also found substantial heterogeneity in the traditional pairwise meta-analysis of Cobb angle, suggesting potential clinical and methodological differences across studies. Heterogeneity may arise from multiple sources, including differences in intervention type, treatment dose and supervision intensity, follow-up duration, patient baseline curve severity, skeletal maturity, and differences in how Cobb angle final values or change scores were extracted across studies (24, 33, 34). Notably, some studies had large effect sizes that may have markedly influenced the pooled result. Therefore, this study did not interpret the pooled SMD as evidence of consistent efficacy across all non-surgical interventions but rather as an overall directional and descriptive result, interpreted jointly with network meta-analysis ranking trends.

The strengths of this study include the systematic integration of RCTs and prospective non-randomized comparative studies around the clinical question of AIS non-surgical treatment, using RCTs as the evidence base for the primary network meta-analysis. This study evaluated both Cobb angle and SRS-22 outcomes, addressing both radiographic changes and patient-reported outcomes. Methodologically, this study distinguished between RCTs and non-randomized comparative studies, assessing risk of bias using the Cochrane risk-of-bias tool and ROBINS-I, respectively, and avoided mixing non-randomized studies with RCTs as the source of primary effect estimates in result interpretation. Furthermore, this study emphasized effect estimates, uncertainty, heterogeneity, and evidence sparsity when interpreting SUCRA rankings, avoiding overinterpretation based solely on ranking probabilities (6, 7).

As suggested by the reviewer, the comparative efficacy of different non-surgical interventions should not be viewed through a single ranking hierarchy. Just as a painter‘s palette contains colors with different purposes, each exercise modality or bracing approach may have specific indications based on patient characteristics, curve pattern, and treatment goals. The SUCRA rankings presented in this study reflect the probability of being best under a specific set of included studies and should not be interpreted as a fixed superiority hierarchy. Furthermore, combination approaches—such as bracing with concurrent Schroth or other scoliosis-specific exercises—may represent the “mixed colors” that yield optimal outcomes for individual patients, although the evidence for such combinations remains limited.

To provide a clinically interpretable framework for the current evidence: (1) For patients with mild-to-moderate AIS (Cobb angle 15°–25°) who are still growing, Schroth-based or other scoliosis-specific exercises may be offered as a first-line adjunctive therapy, with the understanding that evidence for long-term curve control remains limited. (2) For patients with moderate curves (25°–40°) and documented progression risk, bracing remains the best-supported intervention, but its success depends critically on compliance. (3) For curves below 15° or after skeletal maturity with curves below 20°, observation with periodic monitoring is appropriate. The current evidence does not support the superiority of any single intervention across all patient subgroups, and our SUCRA rankings should not be used to mandate specific treatments. This framework is intended to guide clinical reasoning, not to replace individualized decision-making. The cautious interpretation throughout this paper reflects the limitations of the current evidence base—specifically network sparsity, high heterogeneity, and short follow-up durations—rather than any judgment that non-surgical interventions are ineffective.

In summary, this study suggests that bracing and Schroth-related therapies show a trend toward relative superiority for short-term Cobb angle control in AIS, but current evidence remains insufficient to support definitive superiority rankings among different non-surgical interventions. Clinical decision-making should integrate individual patient characteristics, curve progression risk, skeletal maturity, quality of life, compliance, and treatment accessibility. While there is broad consensus that scoliosis-specific exercises should include elements of postural awareness, geometrical derotation (axial elongation), and core stabilization following realignment, the key unresolved question is which specific exercise protocol is most appropriate for which patient phenotype—based on curve magnitude, curve type, skeletal maturity, and compliance profile (22, 24). Our network meta-analysis could not address this question due to the heterogeneity of patient populations and exercise protocols across studies. Future trials should be designed to compare well-defined exercise protocols within more homogeneous patient subgroups and to identify predictors of treatment response.

Future research should prioritize adequately powered, multicenter RCTs with standardized allocation concealment and blinded outcome assessment, standardized intervention protocols, and follow-up until or near skeletal maturity, reporting core outcomes including Cobb angle, curve progression, brace compliance, SRS-22, and adverse events, to improve the reliability and clinical applicability of evidence for AIS non-surgical treatment (22, 24, 30).

## 5 Limitations

This study has several limitations. First, the number of included studies is limited, some intervention nodes are supported by only a few studies, and there is variability across studies in intervention protocols, treatment dose, follow-up duration, baseline Cobb angle, and skeletal maturity, which may affect the stability of the transitivity assumption in the network meta-analysis and lead to imprecision in some comparative estimates. In addition, curves below 20° are often non-progressive, potentially diluting treatment effects, while for curves above 25°, bracing protocols vary substantially across studies in terms of brace type, wearing schedule, and compliance assessment, all of which could not be systematically examined due to the limited number of studies. Second, most studies had short follow-up durations; particularly for bracing, short-term Cobb angle changes may not fully reflect long-term curve progression control effects until skeletal maturity. In addition, several included RCTs had follow-up durations shorter than 6 months. According to clinical guidelines and radiation safety principles, routine repeat radiography for AIS monitoring should not be performed at intervals shorter than 6 months unless clinically indicated (28, 29). Therefore, changes in Cobb angle observed over shorter intervals may reflect natural measurement variability rather than true treatment effects, and their clinical interpretation is limited. This ethical and methodological concern further supports our cautious interpretation of short-term results. Third, evidence for SRS-22 is sparse, and the analysis of patient-reported outcomes should be considered exploratory. Finally, the three prospective non-randomized comparative studies served only as supplementary evidence and could not form the basis for primary conclusions; some RCTs had limitations in reporting randomization, allocation concealment, or blinding, and funnel plot and Egger's regression suggested potential small-study effects. Therefore, the results of this study can provide a reference for comparative efficacy of non-surgical interventions for AIS, but clinical application and interpretation of treatment rankings should be considered cautiously in light of network sparsity, between-study heterogeneity, and risk of bias.

## 6 Conclusion

This systematic review and network meta-analysis suggests that, for AIS patients with Cobb angles below 50°, several non-surgical interventions—particularly bracing and Schroth-based exercises—show trends toward short-term Cobb angle improvement. However, the substantial variability in statistical results reflects the heterogeneity of the interventions themselves rather than evidence against their use. The key challenge moving forward is not to determine a single “best” intervention, but to better define exercise subgroups and their respective indications based on patient-specific factors such as curve magnitude, curve type, skeletal maturity, and compliance potential. Until such stratified evidence becomes available, treatment decisions should be individualized and involve shared decision-making with patients and families.

## Data Availability

All data extracted and analyzed in this study are provided within the manuscript and its Supplementary Material, including the data extraction sheet, risk-of-bias assessments, and statistical analysis dataset. Further inquiries can be directed to the corresponding author.
